# Greenness assessment of a molecularly imprinted polymeric sensor based on a bio-inspired polymer

**DOI:** 10.1186/s13065-024-01313-0

**Published:** 2024-10-22

**Authors:** Hamees A. Adawy, Maha A. Hegazy, Samah S. Saad, Amr M. Bekhet, Shereen A. Boltia

**Affiliations:** 1 Pharmaceutical Analytical Chemistry Department, College of Pharmaceutical Sciences and Drug Manufacturing, Misr University for Science & Technology, 6th of October City, Giza, 16878 Egypt; 2 Pharmaceutical Chemistry Department, Faculty of Pharmacy, Future University in Egypt, Cairo, 11835 Egypt; 3 Analytical Chemistry Department, Faculty of Pharmacy, Cairo University, Kasr Al-Aini Street, Cairo, 11562 Egypt

**Keywords:** Mussel-inspired polymers, Poly (methyldopa), Formoterol, Molecularly-imprinted polymers, Electrochemical sensors, Green analytical procedure index, red green blue model

## Abstract

Methyldopa, a synthesized dopamine substitute with phenolic, amine, and carboxylic groups, was used to create a selective molecular imprinted polymer (MIP) for detecting formoterol fumarate dihydrate (FFD), a long-acting beta2-agonist for asthma and COPD. The bio-inspired polymer (MD) was electro-grafted onto a pencil graphite electrode (PGE) using cyclic voltammetry in a phosphate buffer (pH 6.5). An indirect method involving a redox probe (ferrocyanide/ferricyanide) and differential pulse voltammetry measured FFD binding to the MIP’s 3D cavities. The sensor showed a linear response range from 1 × 10⁻⁹ M to 2 × 10⁻¹⁰ M, with a detection limit of 1.7 × 10⁻¹¹ M. The polymethyldopa (PMD) and FFD interaction was assessed by UV spectroscopy, and the method was validated per ICH guidelines. Green analytical approaches, including RGB and GAPI, were also implemented. The goal was to use advances in molecularly imprinted polymers to develop a more precise and selective electrochemical sensor for FFD quantification.

## Introduction

Under aerobic and alkaline pH conditions, catecholamines have a tendency to be spontaneously oxidised and polymerized by self-assembly, forming a polymeric thin film [1–4], However, the self-polymerization method requires increasing concentrations of monomer and a lengthy time of deposition, also alkaline-labile substrate materials are incompatible with it. An alternate polymerization procedure is operating electro-chemical polymerization techniques such as cyclic voltammetry, the rate of deposition was accelerated at neutral or slightly acidic phase. Dopamine and its substitutes have been shown in numerous investigations to have the capacity to serve as active functional monomers for fabricating electro-polymerized polymer with molecular imprinting (MIP) [4–7], poly(methyldopa) (PMD) has lately attracted interest from the analytical chemistry discipline, owing to having an extra carboxylic group in its structure [8–10], several strategies have been applied for modification of electrochemical sensors with MIPs for increasing selective analysis of various analyte in their dosage form [11, 12] including electro-polymerization, as the electro –active monomers are polymerized when the analyte drug is existing on the electrode cover upon a specified current or voltage is applied [13, 14] which is straightforward accurate, precise, cost effective and analytically quick technique, with no necessity for UV exposure or heating or polymerization initiators, such performance is essentially based on the molecular interaction that happen among the template analyte molecules and the functional monomer. By adjusting the variables related to the number of voltammetric cycles and the scan rate applied, the thickness of the polymeric film and its uniformity and well-adherence properties can be regulated in the experiment.

**Fig. 1:**
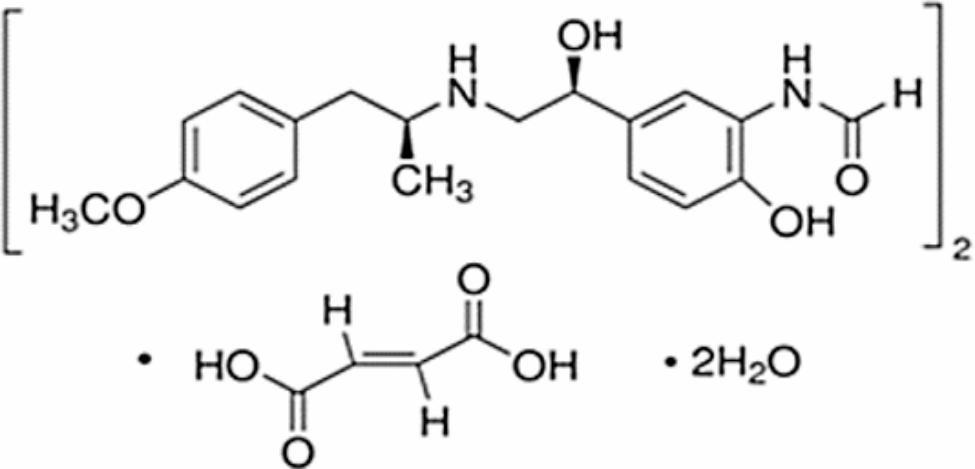
Chemical structure of formoterol fumarate dihydrate

Formoterol fumarate dihydrate (FFD) is chemically known as (2E)-but-2-enedioic acid bis(N-{2-hydroxy-5-[(1R)-1-hydroxy-2-{[(2R)-1-(4-methoxyphenyl)propan-2-yl]amino}ethyl]phenyl}formamide) dihydrate [15] as shown in Fig. 1.

The solubility of the fumarate salt of formoterol in water is 1.16 ± 0.02 mg/mL, soluble in methanol, ethanol and acetonitrile. Formoterol used as long-acting beta2-agonist in the controlling of asthma and chronic obstructive pulmonary disease (COPD). It has similar properties to those of salbutamol, Formoterol fumarate dihydrate is direct acting sympathomimetic, primarily β-adrenoceptor stimulating action that is specific to β2 receptor (β2 agonist). It has rapid onset of action (2–3 min) and has prolonged duration of action up to 12 h, it is utilized in cases when treating reversible airway obstruction, such as chronic asthma or certain COPD patients, requires consistent long acting beta2 agonist treatment. It can be purchased as a single-entity or in various formulations combined with inhaled corticosteroids [15, 16]. A literature survey revealed several UV spectrophotometry methods for the determination of FFD in combination with other drugs. Nasser et al. [17] developed four different spectrophotometric methods for the simultaneous estimation of formoterol with fluticasone, with a linearity range of 2–14 µg/ml for FFD. Aarti et al. [18] developed a spectroscopic determination method for formoterol with mometasone furoate, with a linearity range of 3–8 µg/ml for FFD. Duygu et al. [19] used methyl orange as an ion pair reagent in chloroform for measuring formoterol over the range of 4–20 µg/ml. Shah et al. presented two simultaneous spectrophotometric methods for estimating FFD with beclomethasone over the range of 1–5 µg/ml, applying simultaneous equation and Q absorbance ratio [20]. Boltia S.A et al. [21] developed three different spectrophotometric techniques for the simultaneous estimation of FFD and fluticasone.

Several chromatographic methods have also been reported. Prasanthi et al. achieved the UPLC separation of the drug on C18 with a flow rate of 0.4 ml/min [22]. Narendra et al. [23] established high-performance thin-layer chromatography (HPTLC) for the simultaneous estimation of FFD and fluticasone, densitometrically detected at 215 nm. Rajashi et al. established a stability-indicating HPTLC method with densitometric quantification at 233 nm [24]. Patil et al. developed a stability-indicating HPTLC method with UV quantitation at 281 nm for FFD [25]. Various HPLC techniques [26–32] have also been reported for the simultaneous quantitation of FFD with other drug combinations using different mobile phases and wavelength detections. Finally, a voltammetry method based on square-wave and differential pulse was developed for formoterol in aqueous solution using 0.5 M sulfuric acid, with a linear range of 8 × 10⁻⁶ − 6 × 10⁻⁵ M [33].

All these reported methods have long response times, high solvent consumption, and expensive instrumentation like HPLC and UPLC. In contrast, our suggested procedure is more selective and sensitive, with a concentration estimation range of 1 × 10⁻⁹ M to 2 × 10⁻¹⁰ M, a lower limit of detection and quantitation, and a short response time without requiring extensive sample preparation or extraction steps.

Due to growing of MIPs prominence, being used as smart adsorption particles, the opportunity of manipulating new efficient monomers has extended to cover new substituted molecules with extra functional groups which can bind in a more effective way with particular templates, the bio inspired methyl dopa is responsive to polymerization to form poly-methyldopa (PMD) that have a great attention owed to the prescience of extra carboxylic group (COOH) in its structure, we utilized the bio inspired polymer as a novel electroactive efficient and highly selective monomer to formulate an electro polymerized polymer for the selective quantitation of formoterol fumarate, through the electropolymerization step [34, 35], the purposeful monomers were polymerized as soon as the template drug is existing on the electrode surface, the screening of the interaction between FFD and various functional monomers was performed using UV spectrophotometry measurements to evaluate the plausible molecular interactions between the functional monomer complex and the template, to find out which template monomer had the maximum interaction, the functional monomers o-phenylenediamine, dopamine, and methyldopa were evaluated in order to polymer preparation and hence higher sensitivity, after optimization the method condition the indirect method is applied for the formation of 3D cavities for the selective recognition of the analyte. After washing, the 3D cavities are empty, and redox probes such as ferrocyanide/ferricyanide, can diffuse into the cavities and provide an electrochemical response. Upon rebinding of the analyte of interest, it binds firmly into the 3D cavities after that, the redox probe signal decreases. By the differential pulse voltammetry the calibration curve is constructed with a correlation between the concentration of the template and the reduction of redox probe signal.

Furthermore, the green analytical chemistry principles were evaluated using the Green Analytical Procedure Index (GAPI). This novel tool assesses the environmental impact of the entire analytical process, from sample collection to final estimation. GAPI provides both general and semi-quantitative information using a color-coded system: green for low impact, yellow for medium impact, and red for high environmental impact.

Additionally, we have presented an extra model, called the RGB model, which can be used to appraise analytical methodologies and steps. This model uses three primary colours to estimate three key attributes of the evaluated method: red for analytical performance, green for compliance with green chemistry principles, and blue for productivity/practical effectiveness. By employing these assessment tools, an unbiased and concise evaluation of the analytical methods’ adherence to green analytical chemistry principles can be achieved.

The objective of the current study is to explore methyldopa’s potential as an electro-active monomer for creating an electro-polymerized MIP sensor. This highly selective and sensitive sensor quantitatively determines formoterol fumarate in various matrices using a simple, sensitive, eco-friendly, and time-efficient technique. Various electrochemical factors were evaluated to optimize methyldopa electro-grafting sensitivity. Differential pulse voltammetry (DPV) was used for indirect quantitative estimation of FFD in raw powder and pharmaceutical dosage forms, benefiting routine quality control practices in laboratories.

## Experimental

### Instruments

PalmSens4 potentiostat were be conducted for the electrochemical experiment and operated with PSTrace 5.0 software (Palm Sens, Netherlands), The 0.9 mm diameter pencil graphite electrode (PGE, HB, Rotring, Germany) that serves as the working electrodes, platinum counter electrode, and Ag/AgCl reference electrode for the electrochemical operating cell. For spectrophotometric measurements, a Shimadzu EPMA-1610, Tokyo, Japan, double beam UV-visible spectrophotometer was used. The chemical configuration of the surface was determined using an X-ray photoelectron spectrometer (XPS); (K-Alpha, Thermo Fisher Scientific, WI, USA, X-ray photoelectron spectrometer).

### Materials and reagents

Pure FFD were kindly supplied by Novartis Company Cairo, Egypt. The certified purities were 99.5% ±1.26, according to the reported method [26].

Glacial acetic acid, methanol, dopamine hydrochloride, o-phenylenediamine, methyldopa, potassium ferrocyanide, K_4_[Fe(CN)_6_], and potassium ferricyanide, K_3_[Fe(CN)_6_] were all premium analytical grade chemicals and reagents purchased from Sigma-Aldrich (Darmstadt, Germany). Double-distilled water was gotten from a new human power1 water purifying system (Human Corporation in Seoul, South Korea).

Phosphate buffer (0.1 M) was prepared comprising the pH range from 5.5 to 8.5 as a supportive electrolyte used for electro-polymerization procedure.

#### Pharmaceutical dosage form (Flutiform® inhaler)

Batch number 9h053fc, it is manufactured by Fisons Limited, United Kingdom, and marketing authorization holder: Napp Pharmaceuticals Ltd. (I.A.C. of Mundipharma), Cambridge, United Kingdom. Each metered dose (ex-valve) contains 5.0 µg of (FFD) Formoterol Fumarate Dihydrate and 50.0 µg of drug full name (FP) Fluticasone Propionate.

### Procedure

#### The methyldopa electro –polymerization process

First, the PGE’s bar surface was cleaned from any contaminants by washing it in a methanol and water mixture (1:1v/v). Next, it was dried under a nitrogen stream, then it was connected as a working electrode and immersed in sodium phosphate buffer with pH equal to 6.5 containing 5 × 10^− 3^ M of FFD and 5 × 10^− 3^ M of methyldopa, nitrogen gas was used to purge the solution from oxygen for about fifteen minutes, then ten voltammetric cycles were applied to begin the electro-polymerization process between potential windows from − 0.1 V to 0.8 V using a scan rate of 130 mV/s versus the Ag/AgCl reference electrode.

Subsequently, the formed template was eluted, and the modified electrode, or PMD/PGE, was gently stirred for 20 min with a solution of methanol in addition to glacial acetic acid with the ratio (4:1) v/v while being washed.

Afterwards, the electrode was undergone three consecutive washes with water, then it was subjected to electrochemical characterisation by operating CV in KCl solution (0.1 M) containing an equimolar amount of (5 mM) of [Fe (CN) _6_]^−3/−4^ redox probe, X-ray photoelectron spectrometer (XPS) was employed to characterise the surface chemical configuration of the prepared electrodes. The electrochemical characterization was accompanied by utilizing 0.1 M KCl solution with an equimolar concentration of (5 mM) of [Fe (CN) _6_]^3−/4−^ redox probe.

#### Formoterol fumarate electrochemical measurements

The MIP/PGE electrode was immersed for 10 min in the FFD working solutions with stirring, and then washed with water for 30 s. A solution of 5.0 mM [Fe (CN) _6_]^3−/4−^ in the presence of 0.1 M KCl was measured in phosphate buffer (pH 6.5) by differential pulse voltammetry (DPV) within a potential window of − 0.2 to 0.8 V versus the Ag/AgCl reference electrode at ambient temperature for quantitative estimation of the template.

The calibration curve was constructed by plotting the normalized decrease in the redox probe’s current peak after FFD drug rebinding versus the corresponding template (FFD) concentrations in the range of 2 × 10^− 10^ M to 1 × 10^− 9^ M .

#### Application to pharmaceutical formulation

Flutiform inhaler labelled to contain 5 µg of FFD per one actuation, by taking four actuations in 25-mL volumetric flask, and make further dilution, we reach the final concentration 1 × 10^− 9^ M. Subsequently, the previously described electrochemical measurements were carried out, and drug concentrations were quantitatively estimated using the regression equation that was previously computed.

## Results and discussion

As MIPs become increasingly significant as efficient adsorption particles, there is a scope to use novel functional monomers with extra functional groups to increase their binding capacity to a given template, so increase the selective determination of various analyte in different matrices as dosage form and biological samples, so the electro-polymerization of methyldopa as functional sensing and efficient MIP recognizing material have been implemented. Electro-polymerization, proceeds via applying a specific current or voltage which causes the electroactive monomers to be polymerized instantaneously on the PGE electrode surface in the occurrence of the template FFD analyte, and has the advantage of being simple, highly reliable without requiring a variety of polymerization initiators. Another benefit is that experimental conditions such as the number of applied voltammetric cycles and the scan rate could be adjusted to control the thickness of the polymeric film, resulting in thin, consistent, and extremely adherent films [13].

**Fig. 2:**
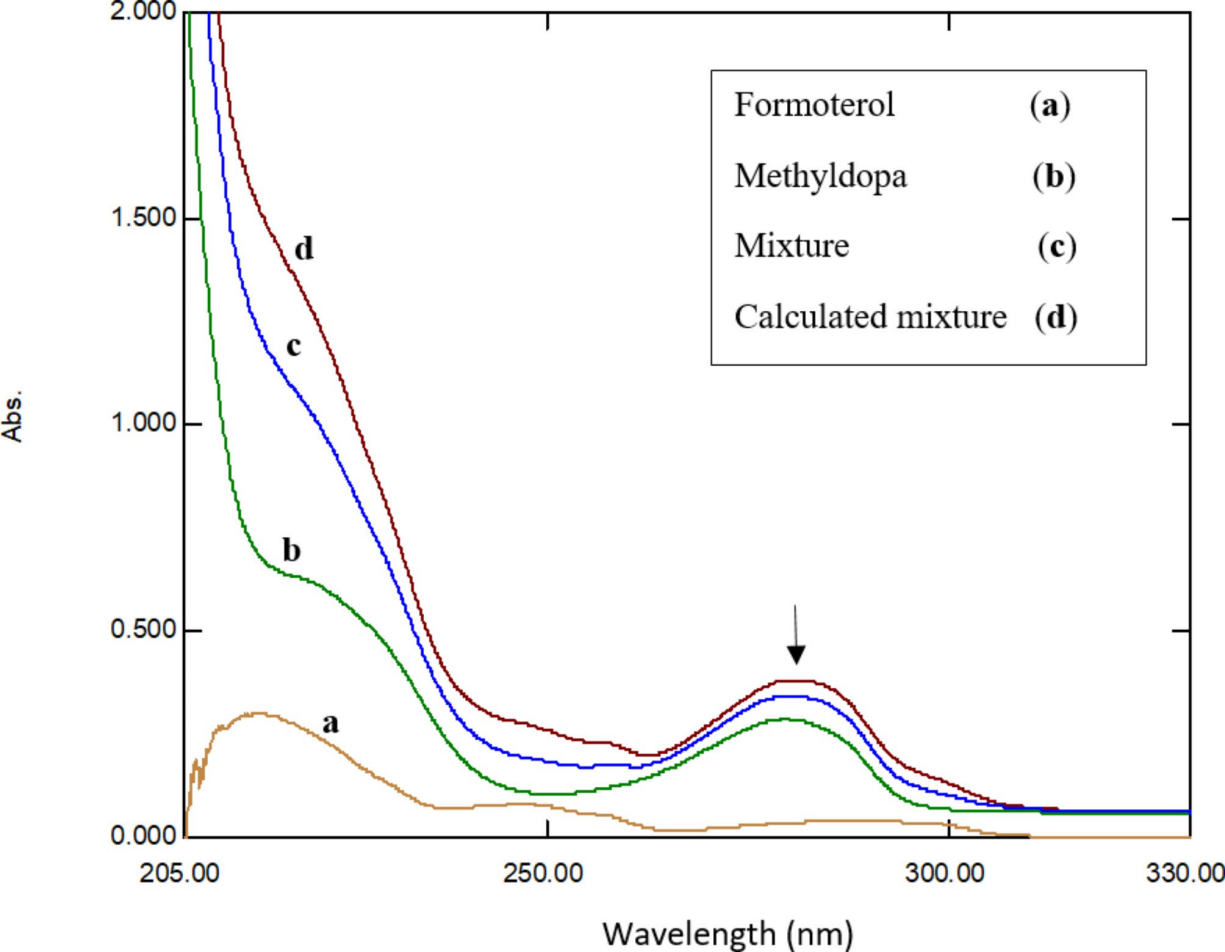
The UV spectra of the template: formoterol fumarate, monomer methyldopa, the equimolar mixture of both, and the calculated sum of formoterol and methyldopa

The conducting of UV spectrophotometric evaluation is an efficient, fast, and economical tool for screening template–monomer complexes [36]. It was observed that the absorption spectrum of the FFD/methyldopa combination shows a marked hyperchromic shift compared to other monomers tried, such as FFD/o-phenylenediamine and FFD/dopamine. As the functionality of polymers imprinted with molecules was mostly dependent on the molecular interactions of analyte molecules and functional monomers created a strongest interaction with the template drug. The FFD/methyldopa UV spectrum represents the creation of a complex with methyldopa characterized by increasing of binding capacity and its stability, as shown in Fig. 2.

### Methyldopa electro-polymerization on PGE surface

Dopamine and its analogues were electro-polymerized in several reported investigations to create polymeric films that worked well as MIPs for the target analyte. We had investigated the electro-polymerization of methyldopa, as a functional monomer, used to fabricate electrochemical sensors of polymers imprinted with molecules, a novel technique was operated and adjusted for methyldopa electro-polymerization via cyclic voltammetric approach, measurements were accomplished on PGEs for their availability, besides being easier to use, more affordable, more cost effective and environmentally friendly than other carbon electrodes [37].

**Fig. 3:**
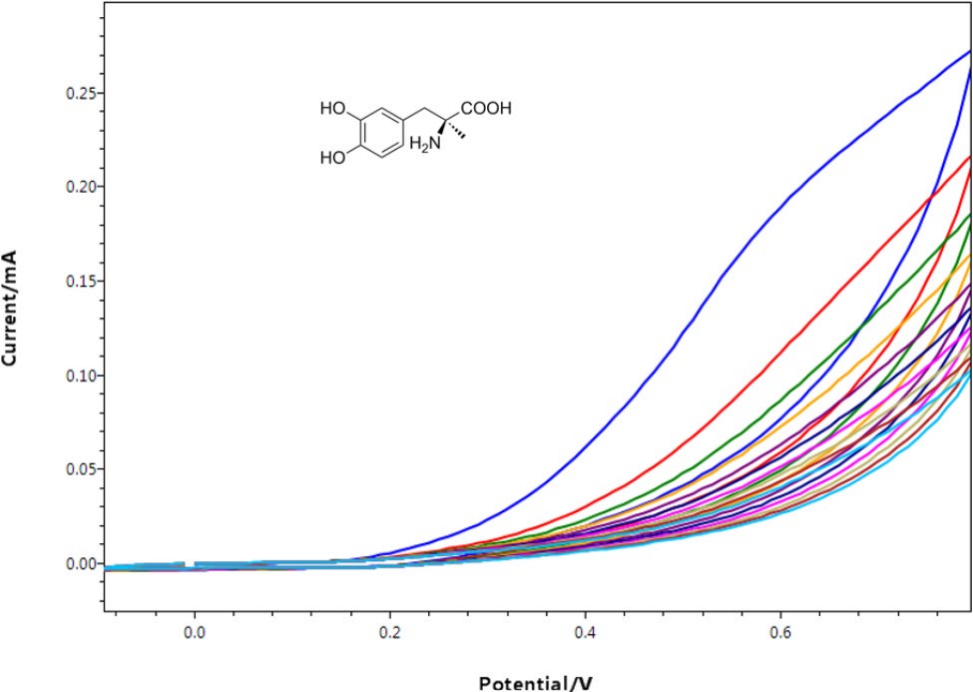
Cyclic voltammograms of electro-polymerization process of methyldopa over 10 cycles with a scan rate of 130 mV/S

The voltammogram of methyldopa electro-polymerization was recorded after 10 cycles of applying a voltage range of -0.1 to 0.8 V at a scan rate of 130 mV/s, can be observed in Fig. 3.

Firstly, molecules of methyldopa were undergone intramolecular oxidation, cyclization, and consequent polymerization to produce a structure resembling melanin. This is indicated by the rapid decline of anodic peak current following the first cycle, which suggests a high rate of electro-polymerization process, that was accomplished without destroying the polymer’s distinct phenolic and carboxylic efficient groups which accountable for additional binding with molecules of FFD [38, 39] Thereafter, as the number of voltammetric cycles increased that the peak current was gradually decreased till it was reduced showing that a PMD polymeric layer had completely covered the PGE surface, and this hinders the electron transfer, PMD/PGEs were formed by electro-polymerization with FFD acting as a template molecule.

#### Electrochemical characterisation for the process of electro-polymerization

**Fig. 4:**
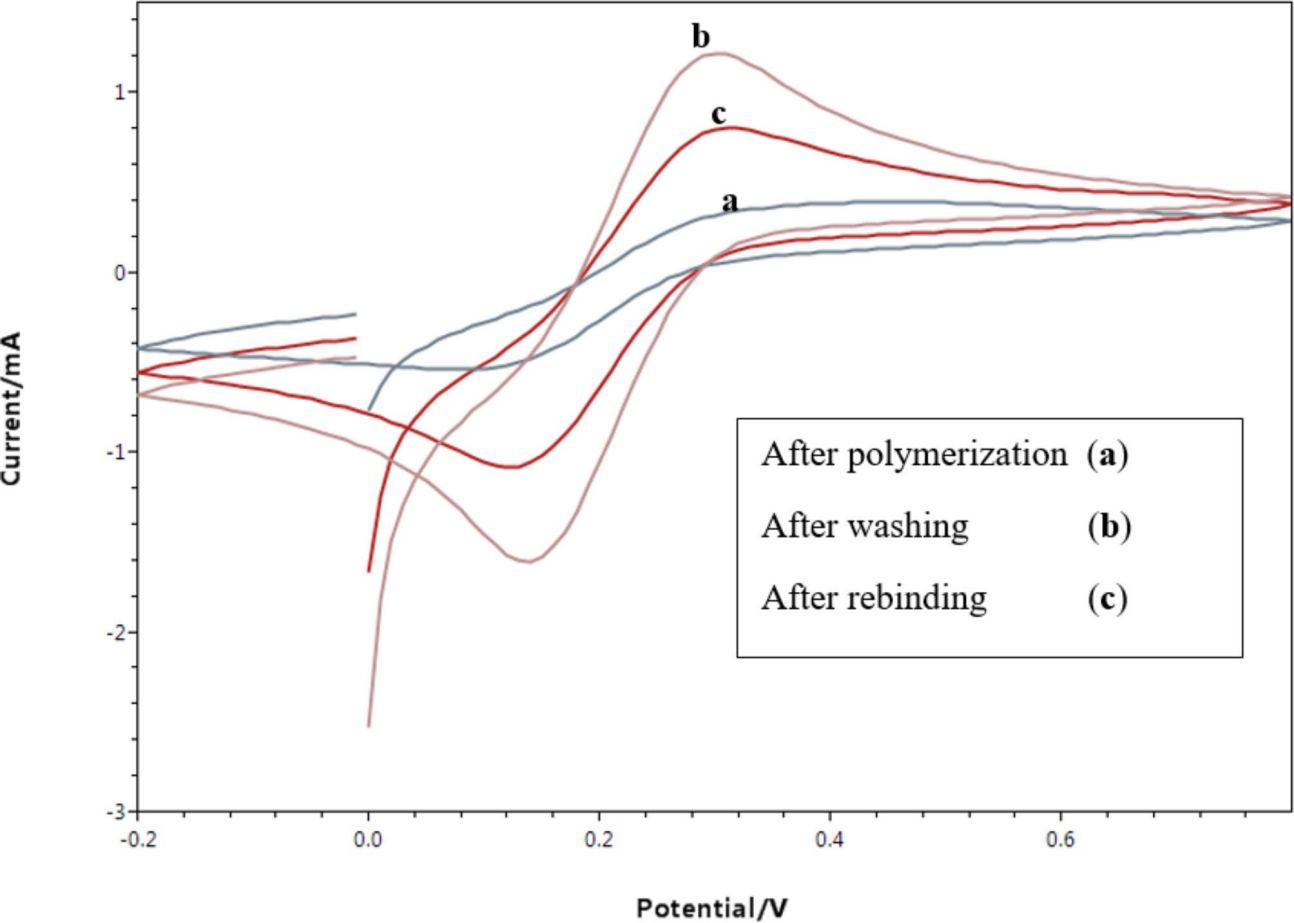
The cyclic voltammograms of the same electrode for a redox probe solution (equimolar 5 mM [Fe (CN) 6]^3−/4−^ in 0.1 M KCl) after polymerization, after washing, and after rebinding

Satisfactory results were obtained when measuring 5 × 10^− 3^ M of FFD in sodium phosphate buffer pH equal to 6.5 by applying voltage over ten cycles utilizing a scan rate of 130 mV/s, subsequently, eluting with a methanol/glacial acetic acid’s mix with a ratio of 4:1 for about 20 min in order to extract the template medication leaving behind three-dimensional channels inside the matrix of polymer, that have function as active pathways for the probe’s transmission, The peak value of the current has obviously reduced at the FFD/PGE surface when the electro-polymerization procedure has finished and prior to template removal. This reflects the PMD coating that insulates the entire PGE surface, preventing electron transmission. Consequently, the probe solution’s redox peaks were successfully established upon rebinding with FFD molecules these channels were blocked with a notable decline of [Fe (CN) _6_]^−3/−4^ signal. These outcomes demonstrated how the imprinted cavity that was created could adsorb the analyte during the rebinding procedure as presented at Fig. 4.

#### X-ray photoelectron spectrometer (XPS) survey

**Fig. 5:**
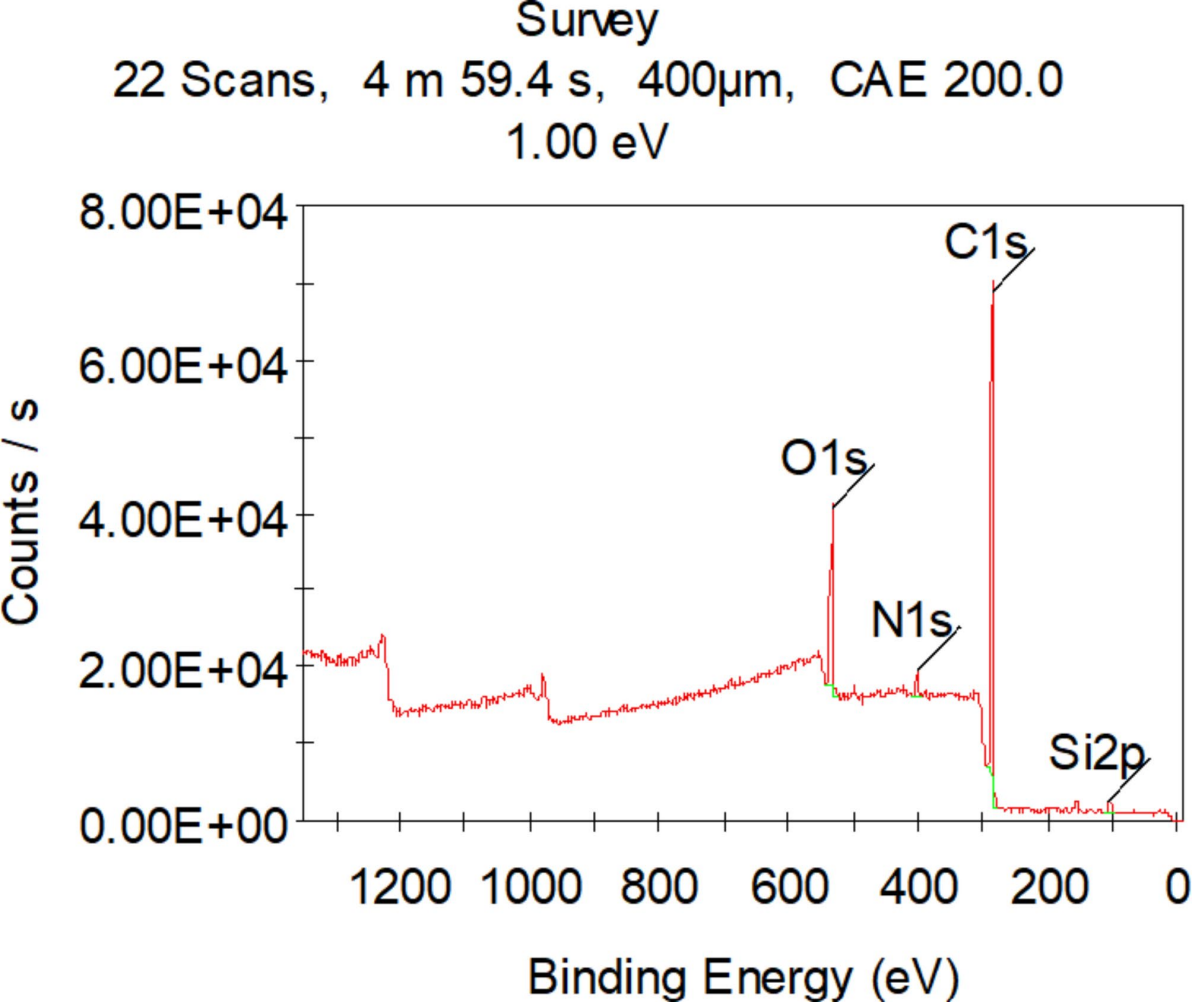
The X-ray photoelectron survey spectrum of the PGE/PMD modified electrode showing three prominent peaks for C 1s, N 1s, and O 1s

Following adherent film deposition on the electrode surface, the characteristics of the film’s chemical and electronic features were examined, Fig. 5 show the PMD/PGE film’s XPS survey spectrum with three distinctive O1s, N1s and C1s peaks, indicating presence of oxygen, carbon and nitrogen comprising group, The successful electro-polymerization of the PMD film uniformly distributed onto the PGE surface was indicated by the appearance of a strong protruding C 1s peak at a binding energy of 285.97 eV, an N 1s peak at 401.16 eV, and an O 1s peak at 533.18 eV. These three peaks confirmed the successful electropolymerization of the bio-inspired polymer onto the PGE surface electrode (PMD/PGE).

#### Condition optimization for measurements of FFD electrochemically using PMD/PGEs

An investigation into enhancing the sensitivity of the sensor involved optimizing experimental conditions. Various parameters such as the number of cycles, scan rate, and pH buffer range were intentionally adjusted using a one-variable-at-a-time approach. The objective was to improve measured outcomes while saving time.

To begin, the template medication was eluted from the cross-linking polymer without altering its structure. A suitable elution solvent was selected, mixed with either an acid or a base to disrupt the electrostatic bonds between the polymer and template, aiming for optimal signal sensitivity and efficient template removal. A mix of methanol and glacial acetic acid was chosen, considering the high solubility of FFD in methanol and the ability of acetic acid to disrupt the hydrogen bonds formed between PMD and FFD [40]. An extraction time of 20 min proved efficient for template removal, and a methanol to acetic acid ratio of 4:1 was identified as satisfactory for achieving reproducible and consistent sensor outcomes.

#### Effect of scan rate

During an investigation into the current response, the scan rate of CV measurements for a 5 × 10^− 3^ M FFD in a sodium phosphate buffer solution was systematically altered, ranging from 70 to 150 mV/s. Notably, the most significant reduction in current response after the washing process and subsequent drug rebinding occurred at a scan rate of 130 mV/s. This finding highlights the sensitivity of the system to changes in the scan rate and underscores the importance of optimizing this parameter for the desired outcomes in the experiment.

#### Effect of pH

The pH of the sodium phosphate buffer solution was investigated alongside CV measurements of 5 × 10^− 3^ M FFD across a range of diverse pH values, ranging from 5.5 to 8.5. The analysis revealed that the most distinct peak formation and consistently reproducible current responses were achieved at a pH of 6.5. This pH value appears to be optimal for obtaining reliable and well-defined outcomes in the CV measurements of the FFD system.

#### Number of cycles

**Fig. 6:**
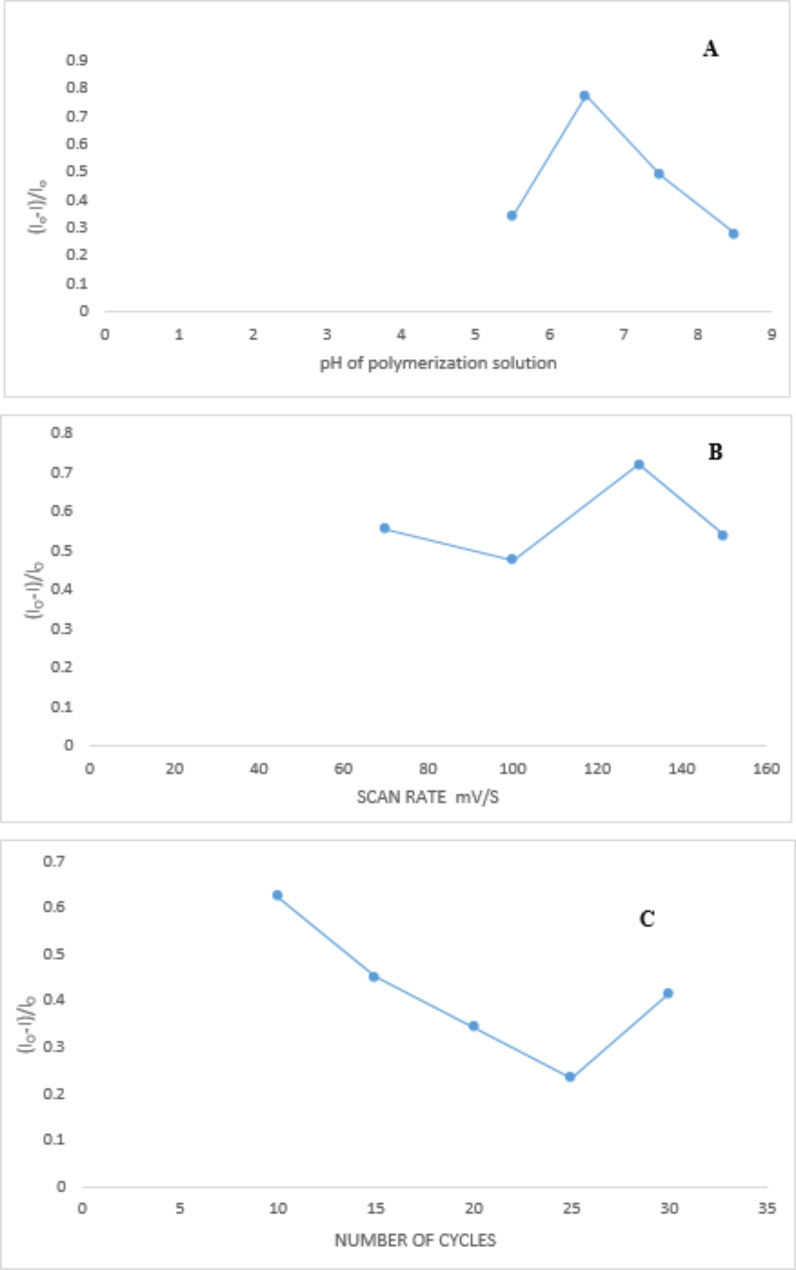
The various parameters optimization that affect the polymerization process of methyldopa (**A**): polymerization pH, (**B**): scan rate, (**C**): number of cycles

The impact of varying the number of cycles on the current response was examined, revealing that the optimal current response was achieved when increasing the number of cycles to ten. Subsequent increases in the number of cycles resulted in a decline in the current response, indicating that further cycles did not contribute to an improvement in sensitivity. The optimization studies played a crucial role in enhancing the sensor’s sensitivity, culminating in a maximum response to FFD, as depicted in Fig. 6.

#### The analytical performance for FFD quantitatively assay by using PMD/PGEs

**Fig. 7:**
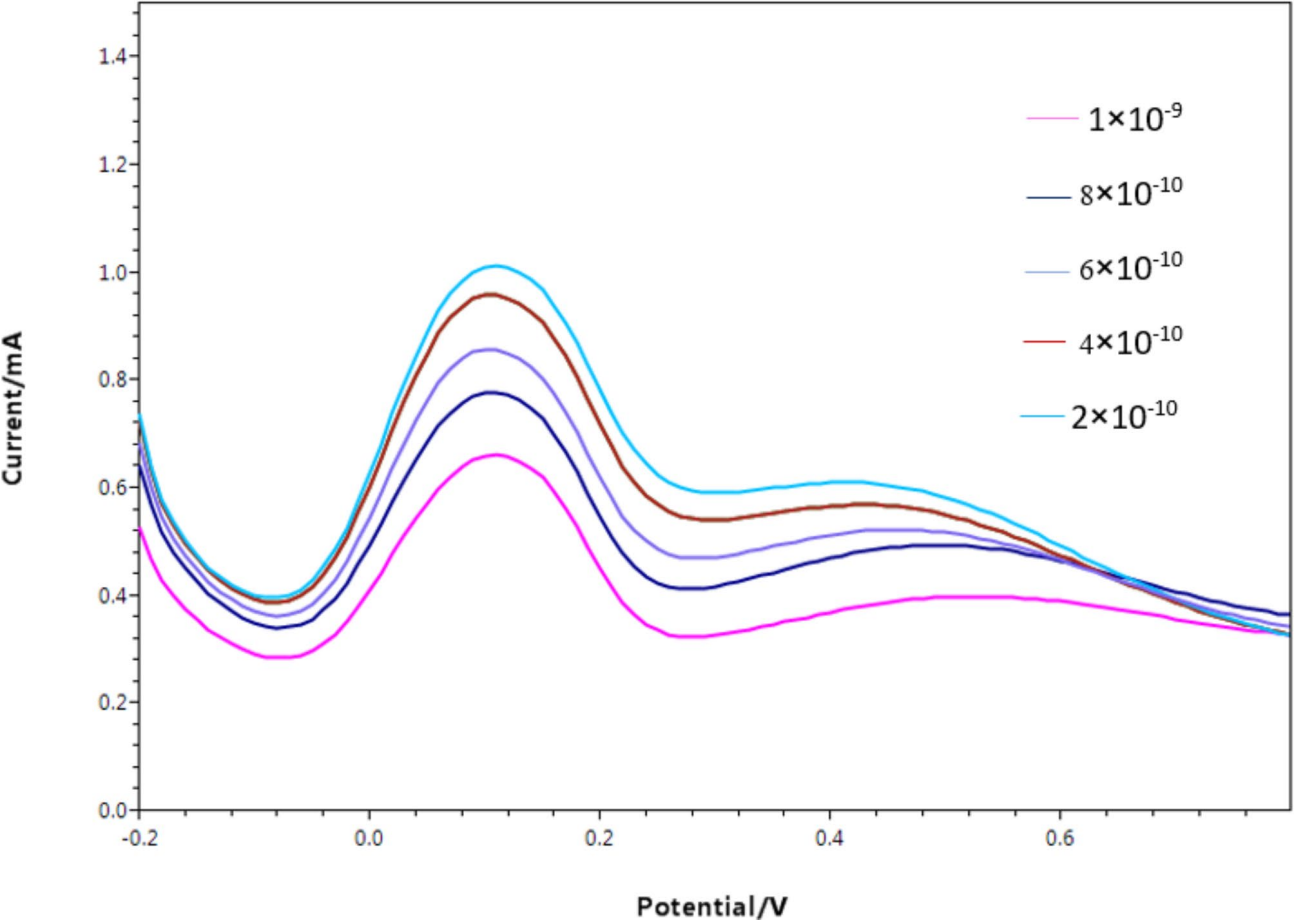
Differential pulse voltammograms of redox probe (equimolar of 5 mM [Fe (CN) _6_]^3−/4−^ in 0.1 M KCl) at MIP/PGE surface in the presence of various concentrations of FFD ranging from (2 × 10^− 10^ M to 1 × 10^− 9^ M) performed at optimum conditions

Differential pulse voltammetry (DPV) was employed instead of CV due to its ability to enhance current sensitivity. This improvement arises from the differentiation between the pulse application and the subsequent decrease in charging current. Optimal signal resolution and a reproducible current response were achieved by using a step potential of 0.01 V, an E pulse of 0.2 V, and a t pulse of 0.02 s, with a scan rate of 0.1 V/s. Subsequently, the current peak responses from DPV measurements were recorded for the redox probe after multiple FFD rebinding events within a concentration range spanning from 2 × 10^− 10^ M to 1 × 10^− 9^ M. This data is visually presented in Fig. 7.

**Fig. 8:**
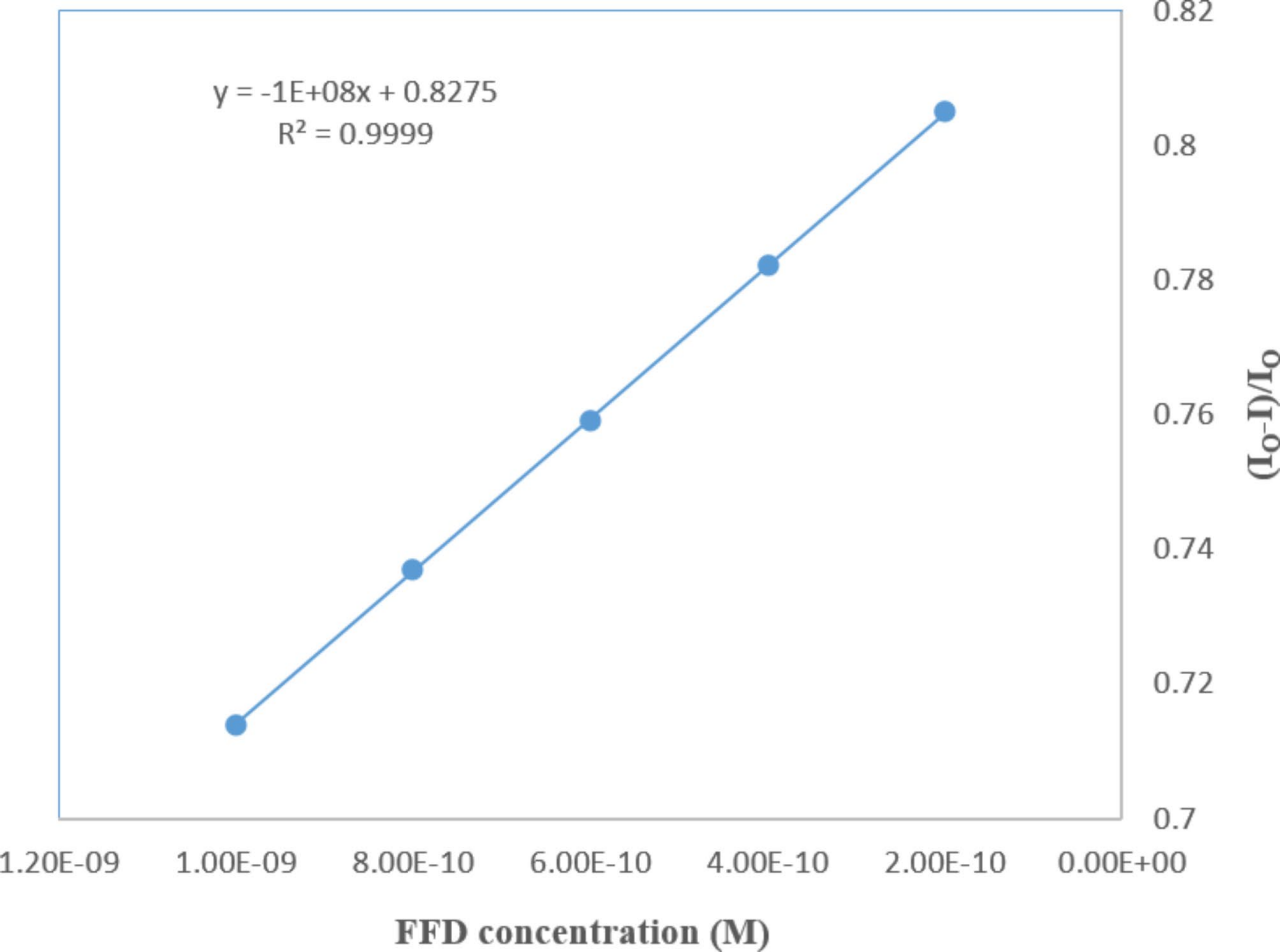
Calibration curve of FFD concentrations ranging from (2 × 10^− 10^ M to 1 × 10^− 9^ M) performed at the optimized conditions

The calibration plot, accompanied by the computed regression equation, is illustrated in Fig. 8.

These figures depict the relationship between the concentration of the template (FFD) and the diminishing signal of the redox probe, providing a comprehensive view of the concentration-dependent response.

### Analytical method greenness profile assessment

Green Analytical Chemistry (GAC) was developed in 2000 with the goal of minimizing or eliminating the adverse effects of analytical procedures on both operators and the environment. Achieving a balance between enhancing the environmental friendliness of an analytical approach and improving the quality of its outcomes is highly challenging. Consequently, finding an appropriate balance between analytical performance and its anticipated greenness is inspiring yet often poses significant challenges for evaluation methodologies, Also the objective of the sustainable development goals of the united nations 2030 agenda is to offset the risk of chemical products and adopting the green chemistry ʼs concepts which aim to develop eco-friendly chemical processes with upgraded human life and environmental quality.

### Green analytical procedure index (GAPI)

**Fig. 9:**
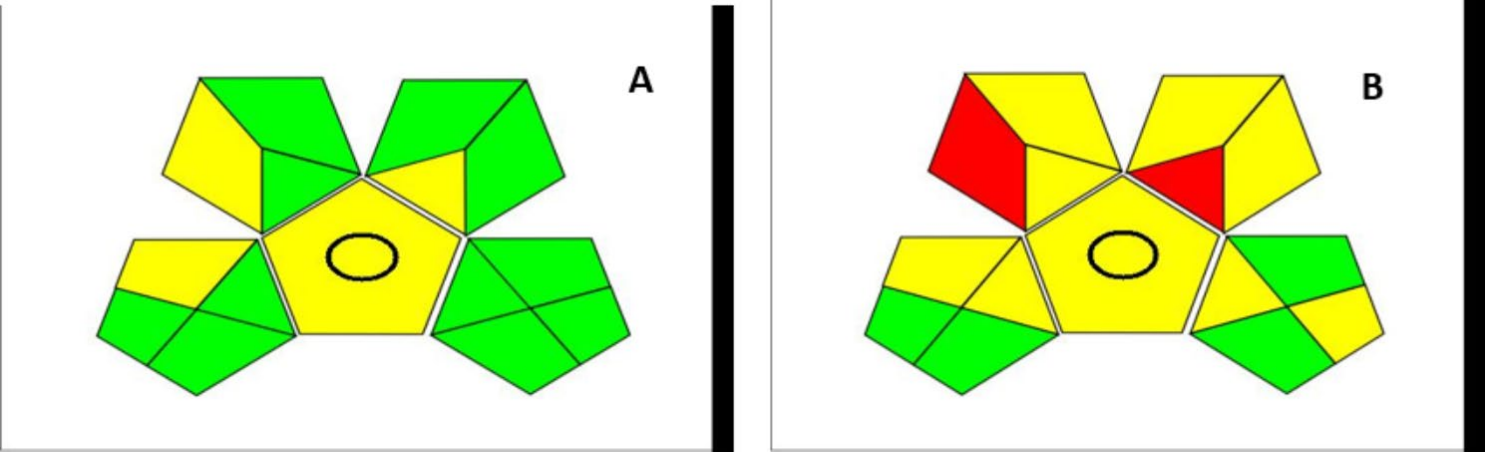
GAPI assessment pictogram generated for the evaluation of proposed method’s greenness (**A**) and the reported method (**B**) ^[25]^

A recently established tool provides semi-quantitative information, enabling individual researchers to assess green criteria according to their own values. It combines the characteristics of both the Eco-scale and NEMI (Płotka-Wasylka, 2018). This technique generates a specific pictogram to rank the ecological influence of each step of the proposed analytical method. It uses five pentagrams to evaluate and measure the ecological impact at each stage, with green, yellow, and red representing low, medium, and high ecological influence, respectively. Each pentagram characterizes a different aspect of the analytical method, such as sample preparation and collection, waste treatment, instrumentation, and the safety of reagents and compounds, including their impact on health. If specific requirements are met, the field is filled green. As a result, this assessment tool is more beneficial than the conventional NEMI tool, which has limitations because its symbol only indicates whether a threat is below or above a specific value [41]. The GAPI pictogram typically displays a satisfactory number of green-shaded sections and fewer, yellow-shaded ones, emphasizing the greenness of the analytical methodology, Fig. 9.

### RGB model (Red, Green and Blue)

The tool’s name derives from the three primary colors (Red, Green, Blue), which correspond to the fundamental aspects of any analytical technique. In this model, analytical performance, evaluated by typical validation procedures, is represented by the red color. Safety and environmental friendliness, encompassing aspects of green analytical chemistry such as waste and reagent hazards, occupational risks, and energy consumption, are represented by the green color. The blue color refers to productivity and practical effectiveness, which includes criteria such as the degree of sample destruction, methodological complexity, cost- and time-effectiveness, and the frequency of instrument maintenance.

The impression of redness, greenness, and blueness is quantitatively measured by a Color Score (CS) ranging from zero to one hundred%. A method reaches one of these primary colors if the corresponding CS is above or equal to 66.6%, a threshold we termed the “satisfaction range.” Conversely, a method loses this primary color if its CS is less than 33.3%, termed the “tolerance range” [42].

**Fig. 10:**
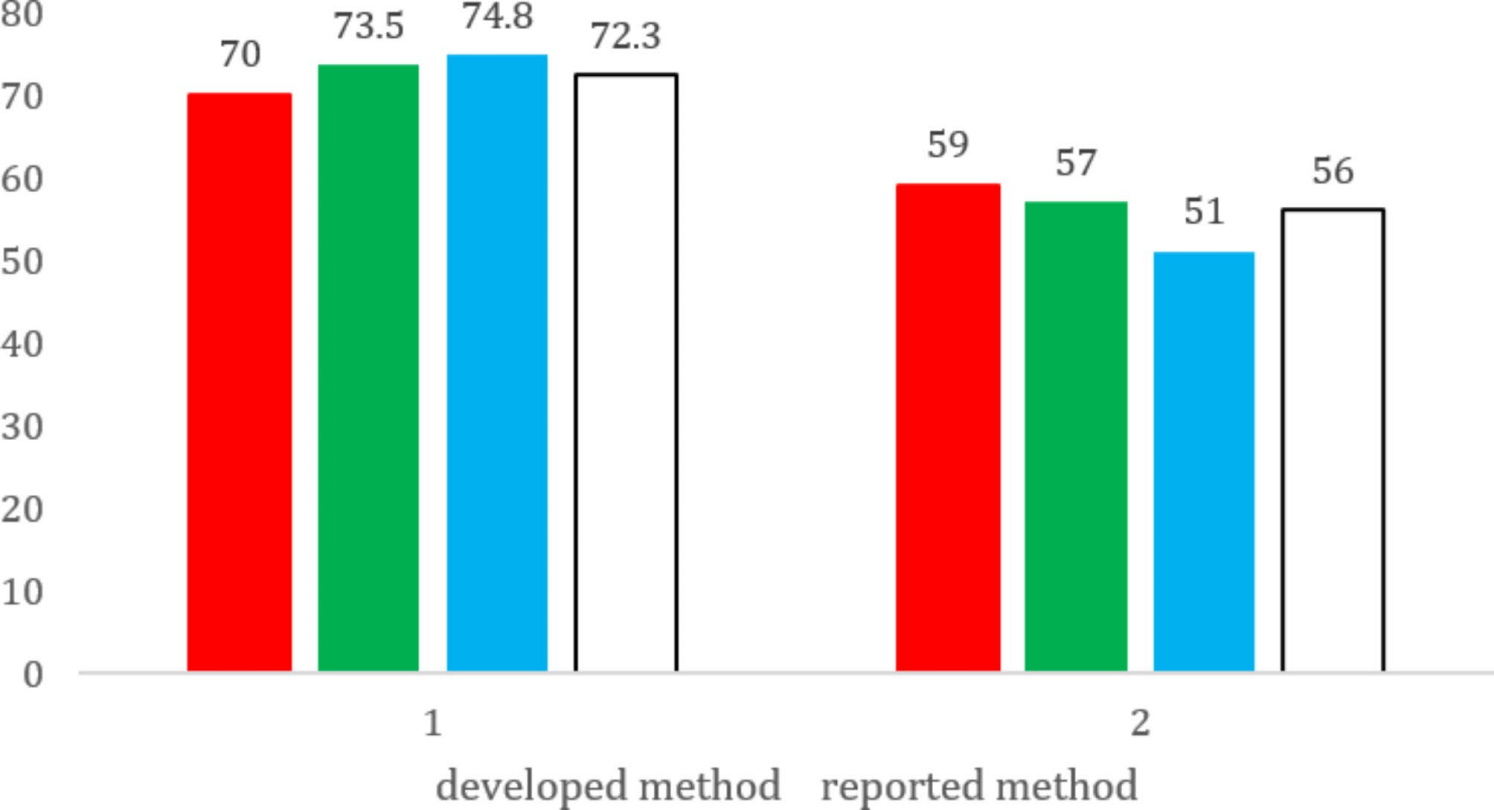
The comparison between the results obtained from RGB model for the developed and reported method

As the usage of methyl dopa monomer has a respectable environmental impact due to the characterized by minimal flammability, toxicity effect and low bioaccumulation effect according to the safety data sheet (SDS) profile, the sodium phosphate buffer has no health or physical hazards with no hazardous decomposition product, and there are no occupational hazard, also the simple preparations of the analytical process utilized green solvents and reagents with miniaturized volumes enhanced the proper implementation of GAC principles that includes: minimizing reagent use, favoring low-toxicity reagents, conserving energy, reducing waste generation, and eliminating hazards to the operator or analyst. Ultimately, the final color achieved after applying the model steps and calculating the method’s brilliance value (73%) is white. This quantitative parameter integrates all aspects of the tool’s criteria, as presented in Fig. 10.

### Method validation

#### Stability and reproducibility of the sensor

The intra-batch and inter-batch reproducibility of the fabricated PMD/PGE sensor were analyzed by evaluating FFD at three concentration levels (2 × 10^–10^, 4 × 10^–10^ and 6 × 10^–10^ M) manipulating three different electrodes within the same day (intra-batch analysis). The same protocol was strictly adhered to ensure consistency.

For intra-batch analysis, the estimated RSD% values are presented in Table 1, demonstrating that they were within the permissible range of less than 2%. This indicates that the suggested method is precise and yields consistent results within the same day.

To evaluate inter-batch reproducibility over three repeated days, the RSD% values, also presented in Table 1, remained within the acceptable range of less than 2%. This further confirms the precision and reproducibility of the method over different experimental days.

Accuracy was evaluated by estimating the recovery percentages for four different concentrations (3 × 10^–10^, 5 × 10^–10^, 7 × 10^–10^ and 9 × 10^–10^ M) falling within the linearity range, as detailed in Table 1. The acceptable recovery percentages obtained affirm the accuracy of the proposed method for FFD analysis.

**Table 1:** Assay validation parameters of the proposed DPV method for the quantitatively determination of formoterol fumarate dihydrate in pure powder form

Parameters (DPV)	FFD
Linearity range (M)	2 × 10^− 10^ M to 1 × 10^− 9^ M
Slope	1E + 08
Standard error of slope	5E + 05
Intercept	0.8275
Standard error of intercept	33E-05
Correlation coefficient	0.9999
Accuracy (mean ± SD)	101.42%±1.08
Repeatability ^a^ (RSD)%	1.026
Intermediate precision ^b^ (RSD)%	1.260
LOD^c^	1.7 × 10^− 11^M
LOQ^d^	5.18 × 10^− 11^ M

^(a)^ Intraday precision; average of three different concentrations of three replicate each (*n* = 9) repeated three times within the same day

^(b)^ Interday precision (*n* = 9) : average of three different concentrations of three replicate each (*n* = 9) repeated on three successive days

^(c)^ LOD (Limit of Detection): 3.3 (SD of residual) / slope

^(d)^ LOQ (Limit of Quantitation):10 (SD of residual) / slope

#### Selectivity of the proposed sensor

The selectivity of the proposed sensor was investigated by evaluating the change in differential pulse voltammetry (DPV) current of the redox probe when measuring 1 × 10^− 9^ M of FFD with the existence of dissimilar interfering drugs at different concentrations. These interfering drugs included salbutamol, a structurally comparable drug used to treat symptoms of asthma and COPD, as well as fluticasone, a co-administered drug with FFD. Additionally, acetaminophen, a commonly co-administered analgesic medication anticipated to be present in-patient plasma, was also assessed. The data provided at Table 2, revealed that the influence of interfering drugs at the FFD peak was insignificant, this observation underscores the high identification, and enhanced fabricated MIP sensor’s selectivity and sensitivity. The sensor has a minimal interference from structurally similar and co-administered medicine, highlighting its specificity for FFD assessment.

**Table 2:** Selectivity evaluation of the fabricated PMD/PGE sensor towards formoterol fumarate (1 × 10^− 9^ M^)^ in the presence of different interfering drugs utilizing DPV technique

Interfering drugs	Concentration(M)	∆I for detection of 1.0 × 10^− 9^ M FFD at 0.11 V	% Relative error in current
Salbutamol	1 × 10^− 9^	0.304	4.10
	5 × 10^− 10^	0.289	3.89
Fluticasone	1 × 10^− 9^	0.217	2.50
	5 × 10^− 10^	0.198	2.28
paracetamol	1 × 10^− 9^	0.076	1.62
	5 × 10^− 10^	0.043	0.90

### Dosage form analysis

The fabricated PMD/PGEs were employed for the quantification of FFD in its marketed dosage form, Flutiform^®^. Three separate samples were analyzed, and the measurements yielded an acceptable average recovery percentage with an RSD percentage of less than 2.0, as detailed in Table 3. This indicates the precision and consistency of the sensor in quantifying FFD in the pharmaceutical formulation.

**Table 3:** Estimation of formoterol fumarate in flutiform^®^ inhaler pharmaceutical dosage form by DPV technique

Preparation	Claimed Concentration	FFD (Recovery %±SD^*^)
**Flutiform** ^®^ **Inhaler** **B.N 9h053fc**	1 × 10^− 9^ M	100.67%± 1.16

*Average of three determinations

Furthermore, standard addition techniques were implemented, and the results, depicted in Table 4, demonstrated appropriate recovery percentages. These findings underscore the heightened affinity for recognition of the MIP sensor toward FFD. The use of standard addition techniques further validates the accuracy and reliability of the sensor for quantifying FFD in complex matrices such as pharmaceutical formulations.

**Table 4:** Application of standard addition technique for the estimation of FFD in pharmaceutical dosage form by the proposed DPV method

pharmaceutical preparation	Added concentration (M)	Taken concentration (M)	Recovery % of FFD
Flutiform ^®^	2 × 10^− 10^	5 × 10^− 10^	99.00
Inhaler	3 × 10^− 10^	5 × 10^− 10^	101.33
B.N 9h053fc	4 × 10^− 10^	5 × 10^− 10^	101.71
	5 × 10^− 10^	5 × 10^− 10^	100.52
**Mean**			**100.62**
**SD**			**1.19**
**RSD%**			**1.18**

Finally, a statistical investigation employing the student t-test and F-test was computed to compare the results gained from the proposed technique and those from a reported HPLC method [26]. With a p-value set at 0.05, it has been found that the computed student t-test and F-test outcomes were lower than their corresponding tabulated ones, indicating a lack of disagreement between the projected and reported process and highlighting the reliability of the fabricated MIP sensor in the quantification of the template (FFD), as illustrated in Table 5.

**Table 5:** Statistical analysis of the results obtained by the proposed DPV methods and the reported HPLC method for the determination of FFD

Parameters	Proposed DPV method	Reported method ^b^ (FFD)
Mean R%	101.62%	99.5%
SD	0.45	1.26
n	5	5
Variance	0.205	1.60
Student’s t- test ^a^	1.75 (2.01)^a^	
F –test ^a^	1.09 (6.38)^a^	

^a^ Tabulated values of t-test and F-test were obtained at *P* = 0.05

^b^ Reported RP-HPLC method using C18 column, acetonitrile: 0.01 m ammonium

Dihydrogen o-rtho phosphate buffer (80:20) v/v as a mobile phase and UV detection at 215 nm [26]

Moreover, the results underwent statistical evaluation using a one-way ANOVA test, revealing no discernible difference between the suggested technique and the reported one, as demonstrated in Table 6. This further supports the consistency and agreement between the proposed MIP sensor method and the established HPLC method for the quantification of FFD.

**Table 6:** Results of one -way ANOVA for comparison of the proposed DPV method for the estimation of FFD and the reported method [26]

	Source of variation	DF	Sum of squares	Mean square	F -value	*P* -value	F crit.
**FFD**	Between groups	1	9.12	9.12	4.63	0.00013	5.32
	Within groups	8	1.575	0.196			
	Total	9	10.69				

## Conclusion

Electrochemical sensors are highly advantageous due to their simplicity, convenience, eco-friendliness, reduced reagent consumption, and cost-effectiveness. They offer rapid analysis with accurate, precise, and reliable results. In this study, an environmentally friendly and sustainable approach was implemented by introducing methyldopa for MIP sensor fabrication. Methyldopa forms a stable complex with FFD through a straightforward electro-polymerization process. The sensor’s chemical composition was defined using XPS. The proposed sensor demonstrated the ability to assess FFD in both bulk and pharmaceutical dosage forms without interference. Its sensitivity in detecting low concentrations in the sub-micromolar range is particularly noteworthy, making it a valuable tool for potential applications in pharmacokinetics research. Furthermore, the interaction between the monomer and the template drug was studied using UV spectrophotometry, showing complex formation that enables the sensor to exhibit excellent selectivity. This allows for the analysis of FFD in the presence of structurally similar and commonly co-administered drugs such as salbutamol and the frequently co-administered painkiller paracetamol. This selectivity positions the sensor as a promising platform for FFD sensing in pharmaceutical formulations and raw materials. Overall, the developed electrochemical sensor presents a versatile and efficient solution for FFD analysis, holding significant promise for various applications in the pharmaceutical field.

## Data Availability

The datasets used and/or analysed during the current study are available from the corresponding author on reasonable request.
